# Accelerating Global Measles and Rubella Eradication—Saving Millions of Lives, Preventing Disability, and Averting the Next Pandemic

**DOI:** 10.3390/vaccines12060699

**Published:** 2024-06-20

**Authors:** David N. Durrheim, Jon K. Andrus, Shahina Tabassum, David Githanga, Mira Kojouharova, Nadia Talab

**Affiliations:** 1 School of Medicine and Public Health, University of Newcastle, Newcastle 2308, Australia; 2 Department of Global Health, Milken Institute of Public Health, George Washington University, Washington, DC 20052, USA; andrusjonkim@yahoo.com; 3 Department of Virology, Bangabandhu Sheikh Mujib Medical University, Dhaka 1000, Bangladesh; 4 Kenya Paediatrics Association, Nairobi P.O. Box 45820-00100, Kenya; 5 National Centre of Infectious and Parasitic Diseases, 1504 Sofia, Bulgaria; 6 Regional Office of Eastern Mediterranean, Cairo 11371, Egypt

**Keywords:** measles, rubella, congenital rubella syndrome, eradication, elimination

## Abstract

No vaccine has been more effective in reducing disease burden, especially in preventing child deaths, than measles-containing vaccine. The return on investment makes measles-containing vaccine one of the most cost-effective public health measures available. Exhaustive reviews of biological, technical, economic and programmatic evidence have concluded that measles can and should be eradicated, and by including rubella antigen in measles-containing vaccine, congenital rubella syndrome will also be eradicated. All World Health Organisation Regions have pledged to achieve measles elimination. Unfortunately, not all countries and global partners have demonstrated an appropriate commitment to these laudable public health goals, and the negative impact of the COVID-19 pandemic on coverage rates has been profound. Unsurprisingly, large disruptive outbreaks are already occurring in many countries with a global epidemic curve ominously similar to that of 2018/2019 emerging. The Immunization Agenda 2030 will fail dismally unless measles and rubella eradication efforts are accelerated. Over half of all member states have been verified to have eliminated rubella and endemic rubella transmission has not been re-established in any country to date. In 2023, 84 countries and areas were verified to have sustained elimination of measles. However, without a global target, this success will be difficult to sustain. Now is the time for a global eradication goal and commitment by the World Health Assembly. Having a galvanising goal, with a shared call for action, will demand adequate resourcing from every country government and global partners. Greater coordination across countries and regions will be necessary. Measles, rubella and congenital rubella syndrome eradication should not remain just a technically feasible possibility but rather be completed to ensure that future generations of children do not live under the shadow of preventable childhood death and lifelong disability.

Fifty years after the Expanded Programme on Immunization was launched in 1974, the extraordinary benefits of life-saving vaccines are being enjoyed by millions of children around the world [1]. No vaccine has been more effective in reducing the disease burden (Figure 1), especially in preventing child deaths, than the measles-containing vaccine (MCV). Between 2000 and 2022, the MCV prevented an estimated 57 million deaths globally [2]. Other than perhaps safe water and sanitation and breastfeeding, no other global health initiative can claim such a remarkable impact in averting deaths. Modeling indicates that measles vaccination will prevent 37% of deaths due to vaccination against 14 key pathogens between 2021 and 2030, with a remarkable 18.8 million lives saved in the current decade alone [3]. In addition to the immediate threats posed by measles disease, the subsequent immunosuppression among children who have been infected may last for weeks to months, leading to increased vulnerability to an array of other viral and bacterial infections [4]. Up to half of all infectious disease-related childhood deaths within the first few years of natural measles infection may be explained by this phenomenon. The return on investment makes the MCV one of the most cost-effective public health measures available. The analysis of economic data from 73 low- and middle-income countries on the costs for delivering the measles vaccine suggests that for every USD 1 invested, USD 58 were saved in future costs from 2001 to 2020 [5].

Congenital rubella syndrome (CRS) is associated with considerable disability, including heart defects, cataracts, hearing loss, and developmental delays, and can occur when a woman is infected with rubella in early pregnancy. The CRS burden is typically low in countries where coverage with rubella-containing vaccines (RCVs) is high and disappears when endemic rubella transmission ceases. Estimates suggest that between 1996 and 2010, the number of children born with CRS decreased from 119,000 to just over 100,000 as the vaccination coverage slowly increased; this is still an intolerable burden of disability given the lower population RCV coverage required to drive this virus to extinction [6]. By including the rubella antigen with the MCV to produce an MR-containing vaccine (MRCV), the elimination of measles will result in the elimination of CRS.

‘Measles can and should be eradicated’ was the conclusion of the Strategic Advisory Group of Experts (SAGE) Working Group on Immunization to the WHO in 2010 following an exhaustive review of biological, technical, economic, and programmatic evidence [7]. By 2010, all WHO regions had pledged to achieve measles elimination, and this was reflected in the Global Vaccine Action Plan, 2011–2020 (GVAP), implying a global measles eradication agenda by default [8]. The GVAP endorsed by all member states simultaneously committed every region to achieving rubella and CRS elimination. Rubella elimination achievement was verified in the region of the Americas in 2015 and measles elimination in 2016.

Unfortunately, not all countries and global partners have demonstrated an appropriate commitment to these laudable public health goals. The devastating global measles resurgence in 2018/2019 bore testament to a failure to achieve the necessary two-dose MCV coverage required to protect all children. Applying the current criteria, the massive and disruptive measles outbreaks in 2019 in many countries met the criteria for the declaration of a Public Health Emergency of International Concern (PHEIC). The World Health Organization (WHO) did not declare it for measles as another PHEIC, COVID-19’s emergence, distracted attention from the measles catastrophe [9]. In contrast with COVID-19, the measles disease burden particularly affects children living in developing countries and so it did not receive the attention and resources devoted to COVID-19, which affected all countries and adult populations. The closure of country borders during the COVID-19 pandemic, coupled with the dramatic decrease in international travel and wide-scale non-pharmaceutical public health measures, significantly obstructed the importation and transmission of measles and rubella viruses and provided an opportunity for accelerated progress towards measles and rubella elimination. However, this would have required concerted and coordinated efforts to close the immunity gaps resulting from the detrimental pandemic impacts on routine immunization program performance and postponed campaigns as a result of COVID-19 [10].

Sadly, only 81% of children born in 2021 received MCV1, the lowest coverage since 2008, leaving 25 million children born that year vulnerable to measles [2]. In 2022, the MCV1 coverage was at 83%, 3% below the pre-pandemic 2019 level of 86%, which had proved inadequate to prevent the epidemic surge at that time. Unsurprisingly, large disruptive outbreaks are already occurring in many countries, with a global epidemic curve ominously similar to that of 2018 and early 2019 (Figure 2), but, additionally, with the knowledge that there is a greater susceptible pool of measles-naïve individuals—both unvaccinated children born since the COVID-19 pandemic and cohorts of older children and young adults who do not enjoy immunity because of historically inadequate routine immunization coverage or sub-optimal campaign performance. It may well be too late to prevent the next measles pandemic.

The Immunisation Agenda 2030 (IA2030) rightly recognizes measles as a tracer of the strength of immunization programs, with strategic priority 3 noting that measles cases and outbreaks should serve to identify weaknesses in immunization programs and guide programmatic planning in identifying and addressing these weaknesses [11]. In reality, IA2030 will inevitably fail unless the measles and rubella eradication efforts are accelerated, including a failure to achieve impact goals 1.1 (deaths averted), 1.2 (endorsed elimination targets), 1.3 (declining trend in number of large or disruptive outbreaks), 2.1 (50% reduction in zero dose children), and 3.1 (90% global coverage with MCV2) [12]. The central tenet of IA2030 is to leave no one behind. As such, the measles virus is the ultimate test of this equity obligation.

Against this bleak backdrop, there are some impressive success stories. Over half of all member states have been verified to have eliminated rubella, and endemic rubella transmission has not been re-established in any country to date (Table 1). This success bodes well for the achievement of global rubella and CRS elimination in the foreseeable future [13], and this progress towards rubella elimination should serve as a catalyst towards measles elimination.

Unfortunately, progress towards global measles elimination has not been homogenous. Only one region has achieved measles elimination, the Americas. This success story has recently been threatened with the loss of ‘measles-free’ status in Brazil and Venezuela due to a sustained, import-related measles outbreak lasting for longer than 12 months. In the European and Western Pacific Regions, 11 countries were verified to have eliminated measles, but, unfortunately, endemic transmission was then re-established (Table 1).

Without a global target, success is difficult to sustain. The other countries of the Region of the Americas have done so, but authorities recognize the fragility of elimination when the virus circulates elsewhere. To this end, there is great promise appearing on the horizon in each region. For example, in the Americas, Brazil and Venezuela appear to have interrupted measles transmission and are likely to apply for reverification. In the Western Pacific, China continues to make phenomenal steady progress towards measles elimination, and the Pacific Island Countries and Areas [14] and Mongolia are targeting 2025 to apply for verification. In the Eastern Mediterranean region, four countries, including Iran and Egypt, have documented and sustained elimination, and five additional countries are close to being verified.

What are the keys to success in this task? Given that each region has committed to measles and rubella elimination, now is the time for a global eradication goal and commitment by the World Health Assembly.

Having a galvanizing goal, with a shared call for action, will demand adequate resourcing from every country government and global partner. However, in addition to health ministers, we need finance ministers appreciating the return on investment. With many countries delegating health responsibility and funding to provincial or local governments, decentralized governments must embrace the goal as well. Such efforts will attract more local engagement and ownership. By proactively looking for opportunities, measles and rubella elimination will enhance national and local health capacity development and the response to future pandemic threats [15].

Champions are needed as advocates in all communities—this should not be too onerous as every community loves its children. New visionary partners are also needed—the world has become too dependent on single major players. Any country not using a rubella-containing vaccine—currently, there are 19—should immediately do so. No scientific arguments justifying the withholding of the RCV exist that have been proven to outweigh the benefits of CRS elimination.

Greater coordination across countries and regions will be necessary. The approach piloted in the Americas remains valid. Coordinated campaigns and surveillance efforts will foster collaboration between countries and achieve more than individual countries working in isolation could ever achieve alone.

Highly effective measles- and rubella-containing vaccines have shown what is possible. With microarray vaccination patches now in clinical trials, this simplified delivery of effective vaccines may guarantee that outreach to every community and child becomes a practical reality [16].

We have no choice—there is a moral imperative to now commit to and achieve measles and rubella eradication [17]. How long will the global community tolerate the ongoing disease burden and preventable deaths due to measles? The rule of rescue demands that if we have effective tools, we should make every effort to save lives. Measles, rubella, and CRS eradication should not remain merely a technically feasible possibility but rather be completed to ensure that future generations of children do not live under the shadow of preventable childhood death and lifelong disability.

## Figures and Tables

**Figure 1:**
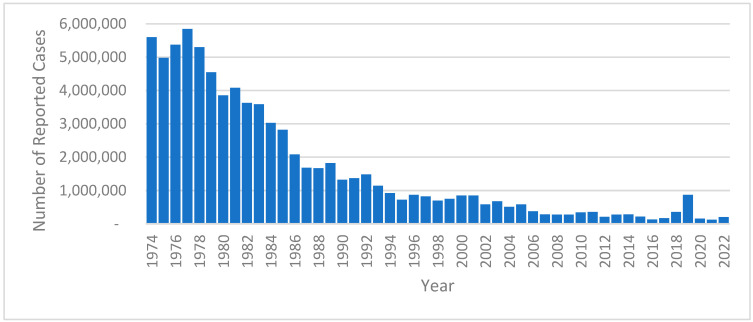
Measles reported cases worldwide, 1974–2022. Source: World Health Organization. Global Health Observatory. Measles—number of reported cases (https://www.who.int/), accessed on 29 April 2024.

**Figure 2:**
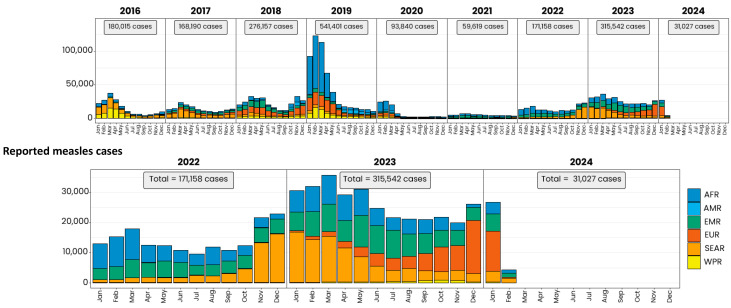
Measles reported cases by WHO region, 2016–2024. Source: World Health Organization, used with permission. Based on data received 2024-02—Data Source: IVB Database. These are surveillance data; hence, for the last month(s), the data may be incomplete. Note: The lower panel is an expanded high-resolution representation of years 2022–2024 in the upper panel. AFR = African Region; AMR = Region of the Americas; EMR = Eastern Mediterranean Region; EUR = European Region; SEAR = Southeast Asian Region; WPR = Western Pacific Region.

**Table 1:** Global measles and rubella elimination verification status by country and territory, 2011–2022.

**Measles Elimination Verification Status by Year**													
**Country**	**WHO Region**	**2011**	**2012**	**2013**	**2014**	**2015**	**2016**	**2017**	**2018**	**2019**	**2020**	**2021**	**2022**
Afghanistan	EMR												
Albania	EUR												
Algeria	AFR												
Andorra	EUR												
Angola	AFR												
Antigua and Barbuda	AMR												
Argentina	AMR												
Armenia	EUR												
Australia	WPR												
Austria	EUR												
Azerbaijan	EUR												
Bahamas, The	AMR												
Bahrain	EMR												
Bangladesh	SEAR												
Barbados	AMR												
Belarus	EUR												
Belgium	EUR												
Belize	AMR												
Benin	AFR												
Bhutan	SEAR												
Bolivia, Plurinational State of	AMR												
Bosnia and Herzegovina	EUR												
Botswana	AFR												
Brazil	AMR												
Brunei Darussalam	WPR												
Bulgaria	EUR												
Burkina Faso	AFR												
Burundi	AFR												
Cabo Verde	AFR												
Cambodia	WPR												
Cameroon	AFR												
Canada	AMR												
Central African Republic	AFR												
Chad	AFR												
Chile	AMR												
China	WPR												
Colombia	AMR												
Comoros	AFR												
Congo, Republic of	AFR												
Cook Islands	WPR												
Country	WHO Region	2011	2012	2013	2014	2015	2016	2017	2018	2019	2020	2021	2022
Costa Rica	AMR												
Côte d’Ivoire	AFR												
Croatia	EUR												
Cuba	AMR												
Cyprus	EUR												
Czechia	EUR												
Denmark	EUR												
Djibouti	EMR												
Dominica	AMR												
Dominica Republic	AMR												
Democratic People’s Republic of Korea	SEAR												
Democratic Republic of the Congo	AFR												
Ecuador	AMR												
Egypt, Arab Republic	EMR												
El Salvador	AMR												
Equatoria Guinea	AFR												
Eritrea	AFR												
Estonia	EUR												
Eswatini (Swaziland)	AFR												
Ethiopia	AFR												
Fiji	WPR												
Finland	EUR												
France	EUR												
Gabon	AFR												
Gambia, The	AFR												
Georgia	EUR												
Germany	EUR												
Ghana	AFR												
Greece	EUR												
Grenada	AMR												
Guatemala	AMR												
Guinea	AFR												
Guinea-Bissau	AFR												
Guyana	AMR												
Haiti	AMR												
Honduras	AMR												
Hungary	EUR												
Iceland	EUR												
India	SEAR												
Indonesia	SEAR												
Iran, Islamic Republic of	EMR												
Country	WHO Region	2011	2012	2013	2014	2015	2016	2017	2018	2019	2020	2021	2022
Iraq	EMR												
Ireland	EUR												
Israel	EUR												
Italy	EUR												
Jamaica	AMR												
Japan	WPR												
Jordan	EMR												
Kazakhstan	EUR												
Kenya	AFR												
Kiribati	WPR												
Kuwait	EMR												
Kyrgyz Republic	EUR												
Lao People’s Democratic Republic	WPR												
Latvia	EUR												
Lebanon	EMR												
Lesotho	AFR												
Liberia	AFR												
Libya	EMR												
Lithuania	EUR												
Luxembourg	EUR												
Madagascar	AFR												
Malawi	AFR												
Malaysia	WPR												
Maldives	SEAR												
Mali	AFR												
Malta	EUR												
Marshall Islands	WPR												
Mauritius	AFR												
Mauritania	AFR												
Mexico	AMR												
Micronesia, Federated States of	WPR												
Monaco	EUR												
Mongolia	WPR												
Montenegro	EUR												
Morocco	EMR												
Mozambique	AFR												
Myanmar	SEAR												
Namibia	AFR												
Nauru	WPR												
Nepal	SEAR												
Netherland, The Kingdom of	EUR												
Country	WHO Region	2011	2012	2013	2014	2015	2016	2017	2018	2019	2020	2021	2022
New Zealand	WPR												
Nicaragua	AMR												
Niger	AFR												
Nigeria	AFR												
Niue	WPR												
North Macedonia	EUR												
Norway	EUR												
Oman	EMR												
Pakistan	EMR												
Palau	WPR												
Panama	AMR												
Papua New Guinea	WPR												
Paraguay	AMR												
Peru	AMR												
Philippines	WPR												
Poland	EUR												
Portugal	EUR												
Qatar	EMR												
Republic of Korea	WPR												
Republic of Moldova	EUR												
Romania	EUR												
Russian Federation	EUR												
Rwanda	AFR												
Saint Kitts and Nevis	AMR												
Saint Lucia	AMR												
Saint Vincent and the Grenadines	AMR												
Samoa	WPR												
San Marino	EUR												
Sao Tome and Principe	AFR												
Saudi Arabia	EMR												
Senegal	AFR												
Serbia	EUR												
Seychelles	AFR												
Sierra Leone	AFR												
Singapore	WPR												
Slovakia	EUR												
Slovenia	EUR												
Solomon Islands	WPR												
Somalia	EMR												
South Africa	AFR												
South Sudan	AFR												
Country	WHO Region	2011	2012	2013	2014	2015	2016	2017	2018	2019	2020	2021	2022
Spain	EUR												
Sri Lanka	SEAR												
Sudan	EMR												
Suriname	AMR												
Sweden	EUR												
Switzerland	EUR												
Syrian Arab Republic	EMR												
Tajikistan	EUR												
Tanzania, United Republic	AFR												
Thailand	SEAR												
Timor-Leste	SEAR												
Togo	AFR												
Tokelau	WPR												
Tonga	WPR												
Trinidad and Tobago	AMR												
Tunisia	EMR												
Türkiye	EUR												
Turkmenistan	EUR												
Tuvalu	WPR												
Uganda	AFR												
Ukraine	EUR												
United Arab Emirates	EMR												
United Kingdom of Great Britain and Northern Ireland	EUR												
United States of America	AMR												
Uruguay	AMR												
Uzbekistan	EUR												
Vanuatu	WPR												
Venezuela ^1^	AMR												
Vietnam	WPR												
Yemen	EMR												
Zambia	AFR												
Zimbabwe	AFR												
Territory/Region	WHO Region	2011	2012	2013	2014	2015	2016	2017	2018	2019	2020	2021	2022
American Samoa (US)	WPR												
French Polynesia (France)	WPR												
Guam (US)	WPR												
Hong Kong SAR (China)	WPR												
Macao SAR (China)	WPR												
New Caledonia (France)	WPR												
Northern Mariana Islands (US)	WPR												
Occupied Palestinian Territories (oPT)	EMR												
Pitcairn Islands (UK)	WPR												
Wallis and Futuna (France)	WPR												
Category	Definition												Code
Endemic	Continuous transmission of measles and/or rubella that persists for ≥12 months in any defined geographical area and no previous verification of elimination.												
Eliminated	Absence of endemic transmission for a continuous period of ≥12 months in the presence of a high-quality surveillance system.												
Verified	Verification of elimination for a region requires that all countries in the region document interruption of endemic virus transmission for a period of ≥36 months.												
Re-established transmission	Presence of a chain of transmission that continues uninterrupted for ≥12 months in a defined geographical area (region or country) after previous verification of elimination.												
No report	National Verification Commission annual report not provided to the Regional Verification Commission for review.												
^1^ (Reverification of Venezuela at the Pan American Health Organization (PAHO)/World Health Organization (WHO) Region of the Americas, Third Annual Meeting of the Measles, Rubella and Congenital Rubella Syndrome Post Elimination Regional Monitoring and Re-Verification Commission held on 14–16 November 2023 and including review of data from first semester of 2023).													
**Rubella Elimination Verification Status by Year**													
Country	WHO Region	2011	2012	2013	2014	2015	2016	2017	2018	2019	2020	2021	2022
Afghanistan	EMR												
Albania	EUR												
Algeria	AFR												
Andorra	EUR												
Angola	AFR												
Antigua and Barbuda	AMR												
Argentina	AMR												
Armenia	EUR												
Australia	WPR												
Austria	EUR												
Azerbaijan	EUR												
Bahamas, The	AMR												
Bahrain	EMR												
Bangladesh	SEAR												
Barbados	AMR												
Belarus	EUR												
Belgium	EUR												
Belize	AMR												
Benin	AFR												
Bhutan	SEAR												
Bolivia, Plurinational State of	AMR												
Bosnia and Herzegovina	EUR												
Botswana	AFR												
Brazil	AMR												
Brunei Darussalam	WPR												
Bulgaria	EUR												
Burkina Faso	AFR												
Burundi	AFR												
Cabo Verde	AFR												
Cambodia	WPR												
Cameroon	AFR												
Canada	AMR												
Central African Republic	AFR												
Chad	AFR												
Chile	AMR												
China	WPR												
Colombia	AMR												
Comoros	AFR												
Congo, Republic of	AFR												
Country	WHO Region	2011	2012	2013	2014	2015	2016	2017	2018	2019	2020	2021	2022
Cook Islands	WPR												
Costa Rica	AMR												
Côte d’Ivoire	AFR												
Croatia	EUR												
Cuba	AMR												
Cyprus	EUR												
Czechia	EUR												
Denmark	EUR												
Djibouti	EMR												
Dominica	AMR												
Dominica Republic	AMR												
Democratic People’s Republic of Korea	SEAR												
Democratic Republic of the Congo	AFR												
Ecuador	AMR												
Egypt, Arab Republic	EMR												
El Salvador	AMR												
Equatoria Guinea	AFR												
Eritrea	AFR												
Estonia	EUR												
Eswatini (Swaziland)	AFR												
Ethiopia	AFR												
Fiji	WPR												
Finland	EUR												
France	EUR												
Gabon	AFR												
Gambia, The	AFR												
Georgia	EUR												
Germany	EUR												
Ghana	AFR												
Greece	EUR												
Grenada	AMR												
Guatemala	AMR												
Guinea	AFR												
Guinea-Bissau	AFR												
Guyana	AMR												
Haiti	AMR												
Honduras	AMR												
Hungary	EUR												
Iceland	EUR												
India	SEAR												
Indonesia	SEAR												
Country	WHO Region	2011	2012	2013	2014	2015	2016	2017	2018	2019	2020	2021	2022
Iran, Islamic Republic of	EMR												
Iraq	EMR												
Ireland	EUR												
Israel	EUR												
Italy	EUR												
Jamaica	AMR												
Japan	WPR												
Jordan	EMR												
Kazakhstan	EUR												
Kenya	AFR												
Kiribati	WPR												
Kuwait	EMR												
Kyrgyz Republic	EUR												
Lao People’s Democratic Republic	WPR												
Latvia	EUR												
Lebanon	EMR												
Lesotho	AFR												
Liberia	AFR												
Libya	EMR												
Lithuania	EUR												
Luxembourg	EUR												
Madagascar	AFR												
Malawi	AFR												
Malaysia	WPR												
Maldives	SEAR												
Mali	AFR												
Malta	EUR												
Marshall Islands	WPR												
Mauritius	AFR												
Mauritania	AFR												
Mexico	AMR												
Micronesia, Federated States of	WPR												
Monaco	EUR												
Mongolia	WPR												
Montenegro	EUR												
Morocco	EMR												
Mozambique	AFR												
Myanmar	SEAR												
Namibia	AFR												
Nauru	WPR												
Nepal	SEAR												
Country	WHO Region	2011	2012	2013	2014	2015	2016	2017	2018	2019	2020	2021	2022
Netherland, The Kingdom of	EUR												
New Zealand	WPR												
Nicaragua	AMR												
Niger	AFR												
Nigeria	AFR												
Niue	WPR												
North Macedonia	EUR												
Norway	EUR												
Oman	EMR												
Pakistan	EMR												
Palau	WPR												
Panama	AMR												
Papua New Guinea	WPR												
Paraguay	AMR												
Peru	AMR												
Philippines	WPR												
Poland	EUR												
Portugal	EUR												
Qatar	EMR												
Republic of Korea	WPR												
Republic of Moldova	EUR												
Romania	EUR												
Russian Federation	EUR												
Rwanda	AFR												
Saint Kitts and Nevis	AMR												
Saint Lucia	AMR												
Saint Vincent and the Grenadines	AMR												
Samoa	WPR												
San Marino	EUR												
Sao Tome and Principe	AFR												
Saudi Arabia	EMR												
Senegal	AFR												
Serbia	EUR												
Seychelles	AFR												
Sierra Leone	AFR												
Singapore	WPR												
Slovakia	EUR												
Slovenia	EUR												
Solomon Islands	WPR												
Somalia	EMR												
South Africa	AFR												
Country	WHO Region	2011	2012	2013	2014	2015	2016	2017	2018	2019	2020	2021	2022
South Sudan	AFR												
Spain	EUR												
Sri Lanka	SEAR												
Sudan	EMR												
Suriname	AMR												
Sweden	EUR												
Switzerland	EUR												
Syrian Arab Republic	EMR												
Tajikistan	EUR												
Tanzania, United Republic	AFR												
Thailand	SEAR												
Timor-Leste	SEAR												
Togo	AFR												
Tokelau	WPR												
Tonga	WPR												
Trinidad and Tobago	AMR												
Tunisia	EMR												
Türkiye	EUR												
Turkmenistan	EUR												
Tuvalu	WPR												
United Arab Emirates	EMR												
Uganda	AFR												
Ukraine	EUR												
United Arab Emirates	EMR												
United Kingdom of Great Britain and Northern Ireland	EUR												
United States of America	AMR												
Uruguay	AMR												
Uzbekistan	EUR												
Vanuatu	WPR												
Venezuela	AMR												
Vietnam	WPR												
Yemen	EMR												
Zambia	AFR												
Zimbabwe	AFR												
Territory/Region	WHO Region	2011	2012	2013	2014	2015	2016	2017	2018	2019	2020	2021	2022
American Samoa (US)	WPR												
French Polynesia (France)	WPR												
Guam (US)	WPR												
Hong Kong SAR (China)	WPR												
Macao SAR (China)	WPR												
New Caledonia (France)	WPR												
Northern Mariana Islands (US)	WPR												
Occupied Palestinian Territories (oPT)	EMR												
Pitcairn Islands (UK)	WPR												
Wallis and Futuna (France)	WPR												
Category	Definition												Code
Endemic	Continuous transmission of measles and/or rubella that persists for ≥12 months in any defined geographical area and no previous verification of elimination.												
Eliminated	Absence of endemic transmission for a continuous period of ≥12 months in the presence of a high-quality surveillance system.												
Verified	Verification of elimination for a region requires that all countries in the region document interruption of endemic virus transmission for a period of ≥36 months.												
Re-established transmission	Presence of a chain of transmission that continues uninterrupted for ≥12 months in a defined geographical area (region or country) after previous verification of elimination.												
No report	National Verification Commission annual report not provided to the Regional Verification Commission for review.												
